# Supplementary material for: The functional anatomy of elephant trunk whiskers
Source: Commun Biol. 2023 Jun 8;6:591. doi: 10.1038/s42003-023-04945-5 (PMC10250425; doi:10.1038/s42003-023-04945-5)
Supplement: Supplementary file 2 — Supplemental Material [file 42003_2023_4945_MOESM2_ESM.pdf]

The functional anatomy of elephant trunk whiskers

Nora Deiringer, Undine Schneeweiß, Lena V. Kaufmann, Lennart Eigen, Celina Speissegger, Ben Gerhardt, Susanne Holtze, Guido Fritsch, Frank Göritz, Rolf Becker, Andreas Ochs, Thomas Hildebrandt & Michael Brecht

Supplementary Figures 1 & 2

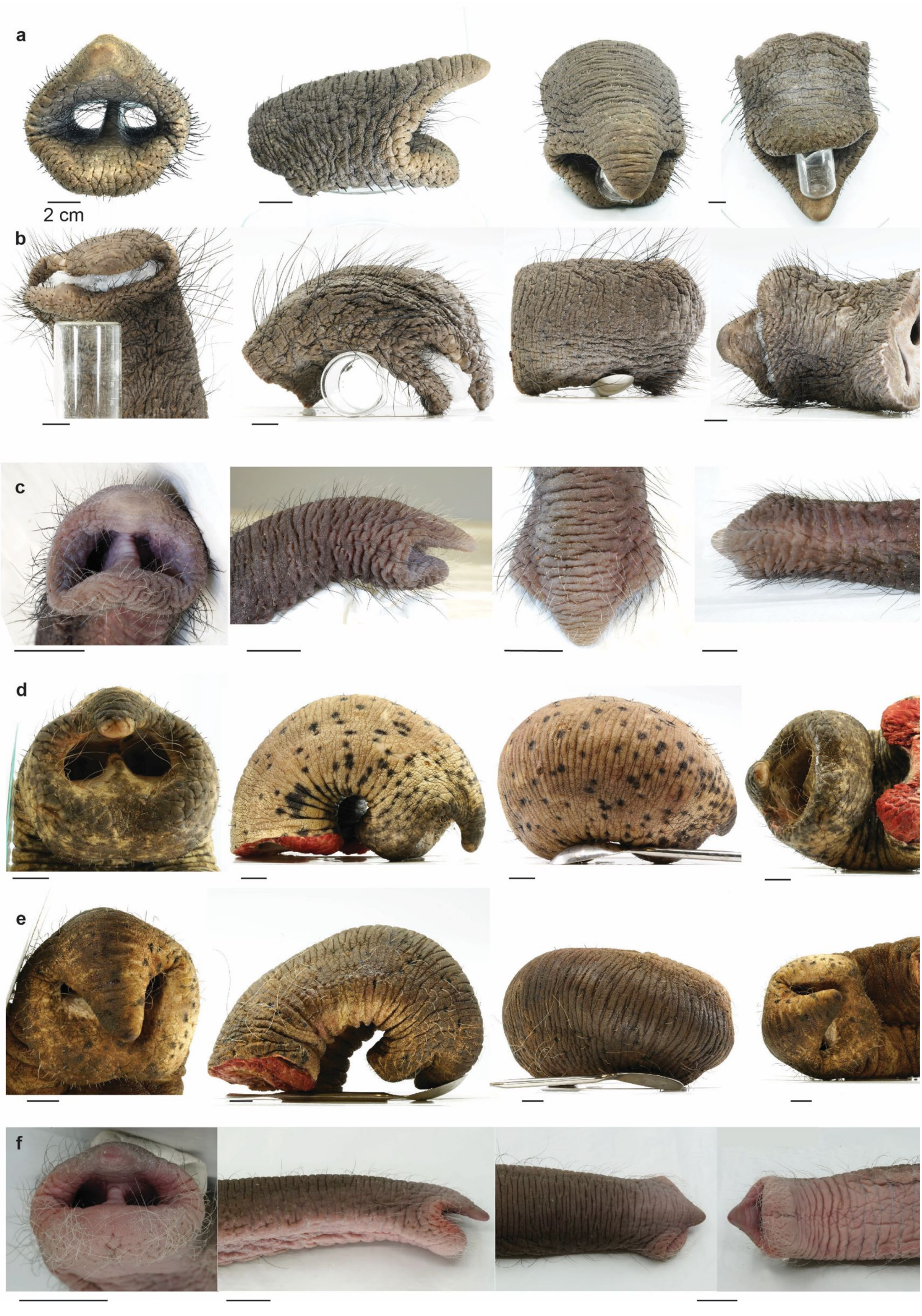
a
2 cm
b
c
d
e
f

Supplementary Figure 1: African and Asian elephant trunk tips.
From left to right in each row: Frontal view of the trunk tip, side view of the trunk tip, view on the dorsal trunk tip side, view on the ventral trunk tip side
a Linda (*Loxodonta africana*, Adult)
b Zimba (*Loxodonta africana*, Adult)
c AM1 (*Loxodonta africana*, Newborn)
d Unknown Asian (*Elephas maximus*, Adult)
e Ilona (*Elephas maximus*, Adult)
f Hoa's Baby (*Elephas maximus*, Newborn)

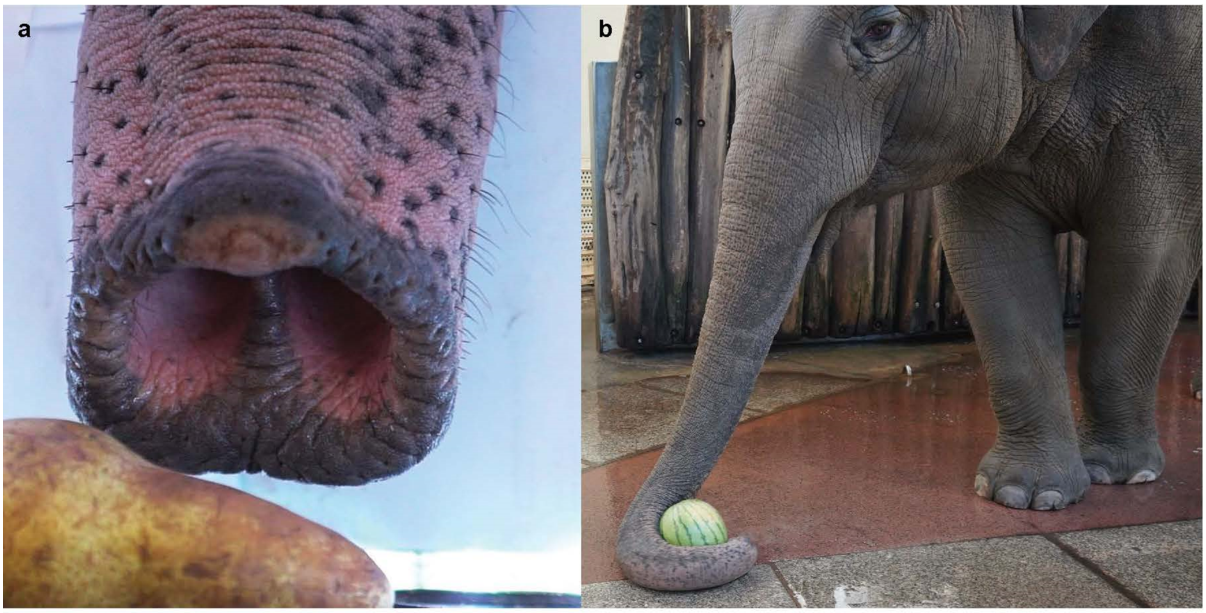

Supplementary Figure 2: Whisker asymmetry is likely caused by lateralized trunk behavior.
a Photograph of the trunk tip of the female Asian elephant Anchali from the Berlin Zoo. Note the asymmetry in whisker lengths.
b Anchali has a pronounced tendency for left-sided grasping, she is a ‘left-trunker’. As shown here for the grasping of a watermelon, such left-sided grasping goes along with the abrasion of right trunk whisker on the ground, i.e., the behavioral asymmetry might lead to lateralized whisker shortening shown in **a**.
