# Supplementary material for: The functional anatomy of elephant trunk whiskers
Source: Commun Biol. 2023 Jun 8;6:591. doi: 10.1038/s42003-023-04945-5 (PMC10250425; doi:10.1038/s42003-023-04945-5)
Supplement: Supplementary file 3 — Description of Additional Supplementary Files [file 42003_2023_4945_MOESM3_ESM.pdf]

Description of Additional Supplementary Files

File name: Supplementary Movie 1

Description: Supplementary Movie 1 shows haptically/ olfactorily controlled pinching of a carrot and vacuuming of an apple.
