# Supplementary material for: The functional anatomy of elephant trunk whiskers
Source: Commun Biol. 2023 Jun 8;6:591. doi: 10.1038/s42003-023-04945-5 (PMC10250425; doi:10.1038/s42003-023-04945-5)
Supplement: Supplementary file 5 — Reporting Summary [file 42003_2023_4945_MOESM5_ESM.pdf]

nature portfolio

Corresponding author(s): Dr. Michael Brecht

Last updated by author(s): Apr 26, 2023

Reporting Summary

Nature Portfolio wishes to improve the reproducibility of the work that we publish. This form provides structure for consistency and transparency in reporting. For further information on Nature Portfolio policies, see our Editorial Policies and the Editorial Policy Checklist.

Statistics

For all statistical analyses, confirm that the following items are present in the figure legend, table legend, main text, or Methods section.

n/a	Confirmed	
☐	☒	The exact sample size (*n*) for each experimental group/condition, given as a discrete number and unit of measurement
☐	☒	A statement on whether measurements were taken from distinct samples or whether the same sample was measured repeatedly
☐	☒	The statistical test(s) used AND whether they are one- or two-sided<br>*Only common tests should be described solely by name; describe more complex techniques in the Methods section.*
☒	☐	A description of all covariates tested
☐	☒	A description of any assumptions or corrections, such as tests of normality and adjustment for multiple comparisons
☐	☒	A full description of the statistical parameters including central tendency (e.g. means) or other basic estimates (e.g. regression coefficient) AND variation (e.g. standard deviation) or associated estimates of uncertainty (e.g. confidence intervals)
☐	☒	For null hypothesis testing, the test statistic (e.g. *F*, *t*, *r*) with confidence intervals, effect sizes, degrees of freedom and *P* value noted<br>*Give P values as exact values whenever suitable.*
☒	☐	For Bayesian analysis, information on the choice of priors and Markov chain Monte Carlo settings
☒	☐	For hierarchical and complex designs, identification of the appropriate level for tests and full reporting of outcomes
☐	☒	Estimates of effect sizes (e.g. Cohen's *d*, Pearson's *r*), indicating how they were calculated

Our web collection on statistics for biologists contains articles on many of the points above.

Software and code

Policy information about availability of computer code

Data collection	Neurolucida Software (MBF Bioscience, Williston, ND)<br>Leica Application Suite X (LAS X, Wetzlar, Germany)
Data analysis	ImageJ (Rasband, W.S., ImageJ, U. S. National Institutes of Health, Bethesda, Maryland, USA) for analysis of pictures, micrographs and videos.<br>Amira software (AmiraZIBEdition 2021, Zuse Institute) for segmentation and analysis of microCT data.<br>The statistical analysis was done using Pythons Numpy (Version 1.20.3), Scipy (Version 1.9.0) and Scikit_posthocs packages (0.7.0).<br>Graphs were compiled using Pythons Matplotlib (Version 3. 4. 3).<br>Photoshop CC 2018 (Version 19. 0. 1) for adjustments of brightness and contrast for pictures.

For manuscripts utilizing custom algorithms or software that are central to the research but not yet described in published literature, software must be made available to editors and reviewers. We strongly encourage code deposition in a community repository (e.g. GitHub). See the Nature Portfolio guidelines for submitting code & software for further information.

Data

Policy information about availability of data

All manuscripts must include a data availability statement. This statement should provide the following information, where applicable:

- Accession codes, unique identifiers, or web links for publicly available datasets
- A description of any restrictions on data availability
- For clinical datasets or third party data, please ensure that the statement adheres to our policy

All data needed to evaluate the conclusions in the paper are present in the paper and/or the Supplementary Materials. Additional data reported in this paper is shared on a publicly accessible repository (https://gin.g-node.org/elephant/Deiringer). This paper does not report original code.

Human research participants

Policy information about studies involving human research participants and Sex and Gender in Research.

Reporting on sex and gender	*Use the terms sex (biological attribute) and gender (shaped by social and cultural circumstances) carefully in order to avoid confusing both terms. Indicate if findings apply to only one sex or gender; describe whether sex and gender were considered in study design whether sex and/or gender was determined based on self-reporting or assigned and methods used. Provide in the source data disaggregated sex and gender data where this information has been collected, and consent has been obtained for sharing of individual-level data; provide overall numbers in this Reporting Summary. Please state if this information has not been collected. Report sex- and gender-based analyses where performed, justify reasons for lack of sex- and gender-based analysis.*
Population characteristics	*Describe the covariate-relevant population characteristics of the human research participants (e.g. age, genotypic information, past and current diagnosis and treatment categories). If you filled out the behavioural & social sciences study design questions and have nothing to add here, write "See above."*
Recruitment	*Describe how participants were recruited. Outline any potential self-selection bias or other biases that may be present and how these are likely to impact results.*
Ethics oversight	*Identify the organization(s) that approved the study protocol.*

Note that full information on the approval of the study protocol must also be provided in the manuscript.

Field-specific reporting

Please select the one below that is the best fit for your research. If you are not sure, read the appropriate sections before making your selection.

[x] Life sciences [ ] Behavioural & social sciences [ ] Ecological, evolutionary & environmental sciences

For a reference copy of the document with all sections, see nature.com/documents/nr-reporting-summary-flat.pdf

Life sciences study design

All studies must disclose on these points even when the disclosure is negative.

Sample size	Sample size was determined by availability of samples. There were a total of n= 13 trunk samples available for external observations of whiskers and n= 4 samples for histological preparations. n= 17 trunks (trunk samples and Zoo animals) were used for observation of whisker length. n= 9 follicle- sinus- complexes (FSCs) from one individual were used for axon count. n= 43 FSCs of one individual were segmented for comparison of lengths. A total of 160 whisker thickness measurements were done on n=4 different trunk samples at different trunk areas.
Data exclusions	No data was excluded.
Replication	Anatomy of the FSC and whisker thickness was verified by using samples from different animals for each species to exclude the possibility of differences being unique to individuals. Two of the whisker counts were redone by a coauthor with -5% and +11% deviation from the previous counts.
Randomization	Randomization was not relevant.
Blinding	Blinding was not possible due to highly obvious anatomical species- differences of the trunk.

Reporting for specific materials, systems and methods

We require information from authors about some types of materials, experimental systems and methods used in many studies. Here, indicate whether each material, system or method listed is relevant to your study. If you are not sure if a list item applies to your research, read the appropriate section before selecting a response.

Materials & experimental systems

n/a	Involved in the study
☐	☒ Antibodies
☒	☐ Eukaryotic cell lines
☒	☐ Palaeontology and archaeology
☐	☒ Animals and other organisms
☒	☐ Clinical data
☒	☐ Dual use research of concern

Methods

n/a	Involved in the study
☒	☐ ChIP-seq
☒	☐ Flow cytometry
☒	☐ MRI-based neuroimaging

Antibodies

Antibodies used	Anti-Neurofilament H Antibody (chicken polyclonal, Millipore, Catalog Nr. AB5539, Lot # 3515590)<br>Goat anti-chicken IgY secondary antibody conjugated to Alexa Fluor 488 (Invitrogen, Catalog Nr. A-11039, Lot # 2304258)
Validation	The Anti-Neurofilament H antibody was controlled by positive control in brain tissue and negative control in kidney and liver tissue by the manufacturer and reacts with Bovine, Human, Mouse, Pig, Rat and Feline tissue as stated on the manufactures website. In a previous study, this antibody was used on elephant tissue producing specific reactivity (Purkart, L., Tuff, J. M., Shah, M., Kaufmann, L. V., Altringer, C., Maier, E., ... & Brecht, M. (2022). Trigeminal ganglion and sensory nerves suggest tactile specialization of elephants. Current Biology, 32(4), 904-910.)

Animals and other research organisms

Policy information about studies involving animals; ARRIVE guidelines recommended for reporting animal research, and Sex and Gender in Research

Laboratory animals	Single whiskers and FSCs were collected from six week old male long-evans rats.
Wild animals	All specimens used in this study came from zoo elephants and were collected by the IZW (Leibniz Institute for Zoo and Wildlife Research, Berlin) over the last three decades.<br>Behavioral experiments with Asian elephants were conducted in the Berlin Zoological Garden.
Reporting on sex	When available, Sex was noted in the table providing an overview of the samples used. Due to the age and different origins of the samples, sex could not be determined in all cases. Sex was not considedered for the analysis, as there were too few samples per species and sex to make effective statements concerning sex differences. From the data available, there were no indications for sex differences.
Field-collected samples	The study did not involve samples collected in the field.
Ethics oversight	Collection of samples by the IZW was in agreement with CITES (Convention on International Trade in Endangered Species of Wild Fauna and Flora) regulations. Specimen reports and CITES documentation for all animals included are held at the IZW. All animals included in the study died of natural causes or were euthanized by experienced zoo veterinarians for humanitarian reasons, because of insurmountable health complications.<br>Our experimental procedures were evaluated by the regional government, which ruled that a formal animal experimentation permit is not required given the non-invasive nature of our procedures (LAGeSo StN-statement 19.07.2021). Behavioral observations were made on several Asian elephants (n = 5), experiments involving training and haptically controlled grasping were performed with the 10-year-old female Asian elephant Anchali.<br>Rat whiskers were collected post mortem from rats killed under a permit approved by the State Office for Health and Social Affairs committee (LAGeSo) in Berlin (Animal license number: G0095-21 / 1.2).

Note that full information on the approval of the study protocol must also be provided in the manuscript.
